# Driving pressure and long-term outcomes in moderate/severe acute respiratory distress syndrome

**DOI:** 10.1186/s13613-018-0469-4

**Published:** 2018-12-07

**Authors:** Carlos Toufen Junior, Roberta R. De Santis Santiago, Adriana S. Hirota, Alysson Roncally S. Carvalho, Susimeire Gomes, Marcelo Brito Passos Amato, Carlos Roberto Ribeiro Carvalho

**Affiliations:** 1 Divisão de Pneumologia, Cardiopulmonary Department, Heart Institute (InCor) University of São Paulo, INCOR Av. Dr. Enéas de Carvalho Aguiar, 44 Pinheiros, São Paulo, SP CEP 05403-900 Brazil; 2 Laboratory of Pulmonary Engineering, Biomedical Engineering Program, Alberto Luiz Coimbra Institute of Post-Graduation and Research in Engineering, Federal University of Rio de Janeiro, Rio de Janeiro, Brazil; 3 Laboratory of Respiration Physiology, Carlos Chagas Filho Institute of Biophysics, Federal University of Rio de Janeiro, Rio de Janeiro, Brazil; 4 Respiratory Intensive Care Unit, University of São Paulo School of Medicine Hospital das Clínicas, São Paulo, Brazil

## Abstract

**Background:**

Acute respiratory distress syndrome (ARDS) patients may present impaired in lung function and structure after hospital discharge that may be related to mechanical ventilation strategy. The aim of this study was to evaluate the association between functional and structural lung impairment, N-terminal-peptide type III procollagen (NT-PCP-III) and driving pressure during protective mechanical ventilation. It was a secondary analysis of data from randomized controlled trial that included patients with moderate/severe ARDS with at least one follow-up visit performed. We obtained serial measurements of plasma NT-PCP-III levels. Whole-lung computed tomography analysis and pulmonary function test were performed at 1 and 6 months of follow-up. A health-related quality of life survey after 6 months was also performed.

**Results:**

Thirty-three patients were enrolled, and 21 patients survived after 6 months. In extubation day an association between driving pressure and NT-PCP-III was observed. At 1 and 6 months forced vital capacity (FVC) was negatively correlated to driving pressure (*p* < 0.01). At 6 months driving pressure was associated with lower FVC independently on tidal volume, plateau pressure and baseline static respiratory compliance after adjustments (*r*^2^ = 0.51, *p* = 0.02). There was a significant correlation between driving pressure and lung densities and nonaerated/poorly aerated lung volume after 6 months. Driving pressure was also related to general health domain of SF-36 at 6 months.

**Conclusion:**

Even in patients ventilated with protective tidal volume, higher driving pressure is associated with worse long-term pulmonary function and structure.

## Background

Acute respiratory distress syndrome (ARDS) is a rapidly progressive illness associated with high mortality and morbidity [1–3]. Up to 5 years after discharge from the intensive care unit (ICU), ARDS survivors still present persistent disabilities, including muscle weakness, altered lung function (e.g., decreased lung volumes, or decreased lung diffusion capacity) and an impaired mental health and cognition [4].

About 25% of ARDS survivors present some reduction in the forced vital capacity (FVC) and in diffusion capacity 6 months after discharge [5]. Among ARDS survivors, abnormal findings in chest tomography correlate with restrictive lung changes and poorer health-related quality of life (HRQoL), suggesting that pulmonary dysfunction could be associated with limited activity in these patients [6].

The risk factors for a reduced long-term lung function in ARDS patients are unknown. As protective mechanical ventilation is an important intervention to reduce mortality of ARDS, probably by decreasing lung inflammation, we hypothesized that the parameters used during the ventilation strategy could be related to long-term lung fibrosis, impairing the lung function among ARDS survivors.

During a recent ARDS trial [7] comparing two strategies of protective mechanical ventilation, we collected lung function data for survivors, during the first 6 months after ARDS onset. We decided to explore the relationship between ventilator settings and long-term outcomes for the entire cohort. Supporting this analysis, we also assessed the acute production of N-terminal-peptide type III procollagen (NT-PCP-III), from enrollment till weaning, as well as other long-term outcomes such as quantitative computed tomography (which enabled us to estimate excess tissue reorganization), 6-min walk test (6MWT) and quality of life (QoL).

## Methods

### Study design

We conducted a prospective longitudinal cohort study of 22 survivors of moderate/severe ARDS, recruited from six different ICUs located in Hospital das Clínicas, São Paulo, Brazil, from November 2008 to January 2012.

### Patient selection

Patients were enrolled in this study in conjunction with a clinical trial in mechanical ventilation, the “ARDSnet Protocol versus Open Lung Approach in ARDS” trial (NCT 00431158) [7]. The institutional review committee approved the study that included the follow-up, and informed consent was obtained from each patient or legal representative. Briefly, this was a randomized controlled trial in which patients were ventilated with a ARDSnet protocol, which uses low tidal volumes, relatively high respiratory rates, with oxygenation managed according to PEEP and FIO2 relationships as defined in a table, or with an open lung approach strategy, which uses a technique to recruit collapsed lung areas and then uses the lowest PEEP level that prevents recollapse of recruited lung units, being the best PEEP level determined by a decremental PEEP trial involving a series of pressure measurements taken after the recruitment maneuver. Both the ARDSnet protocol and the open lung approach require low tidal volumes and plateau pressures. In conclusion, open lung approach improved oxygenation and driving pressure, without detrimental effects on mortality, ventilator-free days or barotrauma.

The inclusion criteria for the study were as follows: Patients intubated and mechanically ventilated, with diagnosis of ARDS using American–European Consensus Criteria and enrollment in study < 48 h since diagnosis of ARDS. For 12–36 h (ideally 12–24 h), after diagnosis of ARDS, patient must be ventilated as follows: volume *A*/*C*, tidal volume of 4–8 mL/kg PBW, plateau pressure ≤ 30 cmH_2_O, PEEP/FIO_2_ adjustments using ARDSnet table, and ventilator rate to keep PaCO_2_ = 35–60 mmHg. During the 12–36-h (ideally 12–24-h) period, PaO_2_/FIO_2_ must remain < 200 mm Hg for an ABG obtained 30 min after placement on the following specific ventilator settings: volume *A*/*C*, tidal volume = 6 mL/kg PBW, plateau pressure ≤ 30 cmH_2_O, inspiratory time ≤ 1 s, PEEP ≥ 10 cmH_2_O, FIO_2_ ≥ 0.5, ventilator rate to keep PaCO_2_ = 35–60 mmHg. No lung recruitment maneuvers or adjunct therapy. Total time on mechanical ventilation < 96 h at time of randomization.

Patients were excluded if they presented one of the following criteria: age < 18 years or > 80 years, weight < 35 kg PBW, body mass index > 60, intubated 2° to acute exacerbation of a chronic pulmonary disease, acute brain injury (ICP > 18 mmHg), immunosuppression 2° to chemo- or radiation therapy, severe cardiac disease (one of the following): New York Heart Association Class 3 or 4, acute coronary syndrome or persistent ventricular tachyarrhythmias, positive laboratory pregnancy test, sickle cell disease, neuromuscular disease, high risk of mortality within 3 months from cause other than ARDS, e.g., cancer, more than 2 organ failures (not including pulmonary system), documented lung barotrauma, i.e., chest tube placement other than for fluid drainage, persistent hemodynamic instability or intractable shock, penetrating chest trauma, enrollment in another interventional study. Randomization in the pivotal study was stratified by center, age and APACHE II scores.

### Measurements

Baseline data collected at enrollment included age, sex, height, severity of illness measured by the Acute Physiology and Chronic Health Evaluation (APACHE) II score, ratio between arterial oxygen tension and fraction of inspired oxygen (PaO_2_/F_I_O_2_) with a positive end-expiratory pressure (PEEP) of at least 10 cmH_2_O and FIO_2_ > 0.5, and static compliance (respiratory system) 30 min before protocol enrollment. Twenty-four hours after patient enrollment, we collected respiratory variables, including tidal volume (mL/kg of predicted body weight), PEEP and plateau pressure. Airway driving pressure was defined as plateau pressure minus total PEEP. To measure the plateau pressure we used neuromuscular blocking agents and volume-controlled ventilation.

Blood samples were obtained on the day of randomization (day 0) and on days 1, 3, 7, and the day of extubation. Blood samples were centrifuged, and plasma was stored at 70 C. NT-PCP-III was assayed by a sandwich ELISA method according to the methodology specifications of the manufacturer (Elabscience, Texas, USA). The normal range of serum levels of nonsmoking individuals was determined to be 1.6–4.0 ng/L.

Pulmonary function testing (PFT) was performed at 1 and 6 months after the onset of ARDS using the MedGraphics Cardiorespiratory Diagnostic System (Medical Graphics Corporation, USA). All tests were done according to Brazilian guidelines [8]. Reference ranges were calculated based on statistics formulated from the Brazilian population [9–11].

High-resolution computed tomography (HRCT) scan of the lungs was also performed 1 and 6 months after the onset of ARDS in supine position during inspiration, close to total lung capacity. All CT scans were segmented by applying the region growing algorithm to select the lung parenchyma using OsiriX (OsiriX 64-bits, Pixmeo Sarl, Geneva, Switzerland). After the segmentation, the original images (DICOM files) as well as each respective ROI were exported and analyzed with a purpose-built routine (QALI-DV software) written in MATLAB (MathWorks, USA). Manual correction was applied to the segmented images containing peripheral atelectasis. Total lung volume (TLV), total air volume (TAV) and total lung mass (TLM) were extracted from the segmented whole lung in 3D [11, 12]. The percentages of hyperaerated (− 1000 to − 900 HU), normally aerated (− 900 to − 500 HU), poorly aerated (− 500 to − 100 HU) and nonaerated (− 100 to + 100 HU) compartments of the lung parenchyma were calculated [12, 13]. To assess the occurrence and progression of emphysema in this longitudinal study we used the percentile point [14–17] using a threshold of 15% (P15). The sensitivity of the percentile point method has been shown to be similar within a broad range of percentiles from the 10th to the 30th [14].

Six months after ARDS onset, we also performed a standardized 6MWT [18] and fulfilled the Medical Outcomes Study 36-item Short-Form General Health Survey (SF-36), which measures the HRQoL [19]. The SF-36 includes eight multiple-item scales that assess physical functioning, social functioning, physical role, emotional role, mental health, pain, vitality and general health. Scores for each aspect can range from 0 (worst) to 100 (best).

### Statistical analysis

Categorical values are described as frequency and percentages, and continuous variables as the mean and SD, or median and interquartile range. The Fisher exact test was used to compare independent categorical variables. Continuous variables were compared with the Student *t* test or the Mann–Whitney test for dependent or independent data. The Friedman test was used for one-way repeated-measures analysis. The strength of the association between two variables was measured using correlation coefficient (*r*). We used Pearson correlation to parametric variable and Spearman correlation to nonparametric variable. We used linear regression to get *r*^2^ in parametric variable. In order to determine clinical variables independently associated with lung function, we performed a multivariable linear regression. For NT-PCP-III regression we used log10 transformations as is commonly performed for biomarkers with a right-tailed distribution. Statistical significance was set at a two-tailed *p* value of ≤ 0.05, and analyses were performed with *R*, version 3.0.2 (http://www.r-project.org).

## Results

### Characteristics of the population

**Fig. 1:**
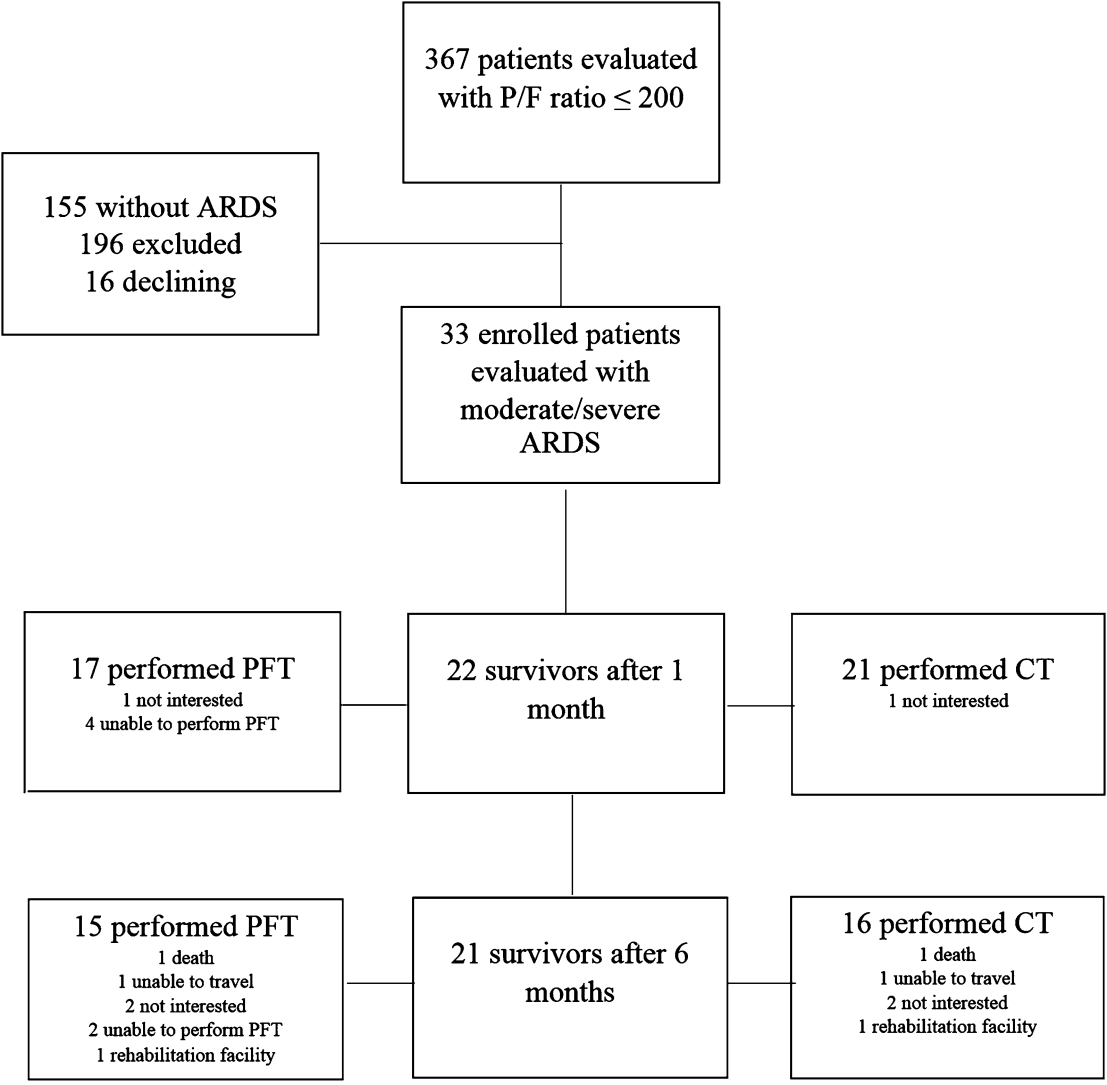
Chart of the protocol

Over the 58-month (2007–2012) recruitment period, we enrolled 33 patients. The patients in cohort were predominantly male. Mortality at 28th day was 33% (Fig. 1).

Of those enrolled, we lost the follow-up of 5 patients at month-1 and 2 between month-1 and month-6. Reasons for exclusion are outlined in Fig. 1. There was no difference in terms of severity score, age and static compliance measurement between patients followed up at month-6 and patients that were lost.

The descriptive baseline and hospital data for all randomized patients and for survivors are described in Table 1. Monitored variables during mechanical ventilation at baseline and 24 h after randomization are also shown in Table 1. Driving pressure at 24 h was related to driving pressure at 48 h (*r* = 0.58, *P* = 0.006) and 72 h (*r* = 0.56, *P* = 0.009) after randomization.

**Table 1:** Demographic characteristics and mechanical ventilation variables from randomized and surviving ARDS patients

Characteristics	Randomized patients (*N* = 33)	Surviving patients (*N* = 22)	Nonsurviving patients (*n* = 11)	*P* value
Age, year	49 ± 14.9	48.5 ± 13.9	50 ± 16.7	0.80
Sex, % male	22 (66.7)	15 (68)	7 (46.7)	1.00
Smokers (%)	10 (30)	7 (28)	3 (27)	1.00
Origin of ARDS				
Primary N, %	23 (70)	17 (77)	6 (55)	0.24
Secondary N, %	10 (30)	5 (23)	5 (45)	
Baseline data				
APACHE II	19.6 ± 10.5	17.1 ± 5.3	24.5 ± 16.0	0.16
*P*/*F* ratio	129 ± 32	135 ± 34	118 ± 23	0.09
Tidal volume, mL/kg PBW	5.9 (5.7–6.0)	5.9 (5.5–6.0)	5.9 (5.9–6.4)	0.78
Driving pressure, cmH_2_O	13.5 ± 4	13.2 ± 3.9	14.1 ± 4.3	0.58
Plateau pressure, cmH_2_O	25.4 ± 3.8	25.3 ± 4.2	25.6 ± 3.3	1.00
PEEP, cmH_2_O	10 (10–14)	10 (10–14)	10 (10–13)	0.69
*C*_stat_, mL/cmH_2_O/kg PBW	0.48 ± 0.15	0.47 ± 0.15	0.50 ± 0.16	0.69
Data 24 h after inclusion				
OLA arm patients, %	18 (54%)	11(50%)	7 (63%)	0.71
*P*/*F* ratio	173 ± 62	174 ± 57	170 ± 70	0.96
Tidal volume, mL/PBW	5.3 ± 1.1	5.5 ± 0.9	4.9 ± 1.4	0.20
Driving pressure, cmH_2_O	11 (10–14)	11 (10–14)	12 (10–12.5)	0.92
Plateau pressure, cmH_2_O	28 (26–30)	27.5 (26.2–30)	30 (24.5–31)	0.48
PEEP, cmH_2_O	15.4 ± 5.1	15.3 ± 4.9	15.5 ± 5.5	0.93
*C*_stat_, mL/cmH_2_O/kg PBW	0.44 (0.40–0.53)	0.45 (0.41–0.54)	0.43 (0.37–0.47)	0.19
Days of ventilator use	9 (6.5–13.5)	9 (6.2–11.7)	12 (10–14.5)	0.14
ICU length of stay, days	16 (11.7–24)	18.5 (12–24.7)	15 (12.5–17)	0.26
Hosp. length of stay, days	27.5 (16.7–56.7)	34 (20.7–73.5)	21 (15–25)	0.02

Parametric data are presented as mean ± 1 standard deviation or median (first and third quartiles)

*P/F ratio* PaO_2_/FIO_2_ ratio, *PBW* predicted body weight and *C*_*stat*_ static compliance

### Pulmonary function tests analysis

Pulmonary function tests showed a mildly reduced FVC and a moderately reduced DL_CO_ after 6 months (Table 2). The FVC was below normal (< 80% of predicted) in eleven (65%) patients at month-1, and in five (33%) patients at month-6. Twelve patients (70%) showed a reduced DL_CO_ at month-1, whereas four (29%) patients showed a reduced DL_CO_ after 6 months of follow-up (Table 2).

**Table 2:** Lung function during the follow-up

	1 Month (N = 17)	6 Months (N = 15)
FVC (L)	3.34 ± 0.77	3.78 ± 1.11
FVC (% predicted)	80 ± 16	89 ± 17
FEV1/FVC ratio	0.81 ± 0.05	0.78 ± 0.06
FEV1/FVC ratio (% predicted)	99 ± 5	97 ± 8
TLC (L)	4.96 ± 1.18	5.57 ± 1.36
TLC (% of predicted)	82 ± 17	89 ± 18
RV (L)	1.71 ± 0.55	1.75 ± 0.49
RV (% predicted)	98 ± 27	97 ± 21
DLco	17.8 ± 6.1	24.0 ± 8.1
DLco (% predicted)	55 ± 17	71 ± 17

All data are presented as mean ± 1 standard deviation

*FVC* forced vital capacity, *FEV1* forced expiratory volume in 1 s, *TLC* total lung capacity, *RV* residual volume and *DLco* diffusing capacity of the lung for carbon monoxide

**Fig. 2:**
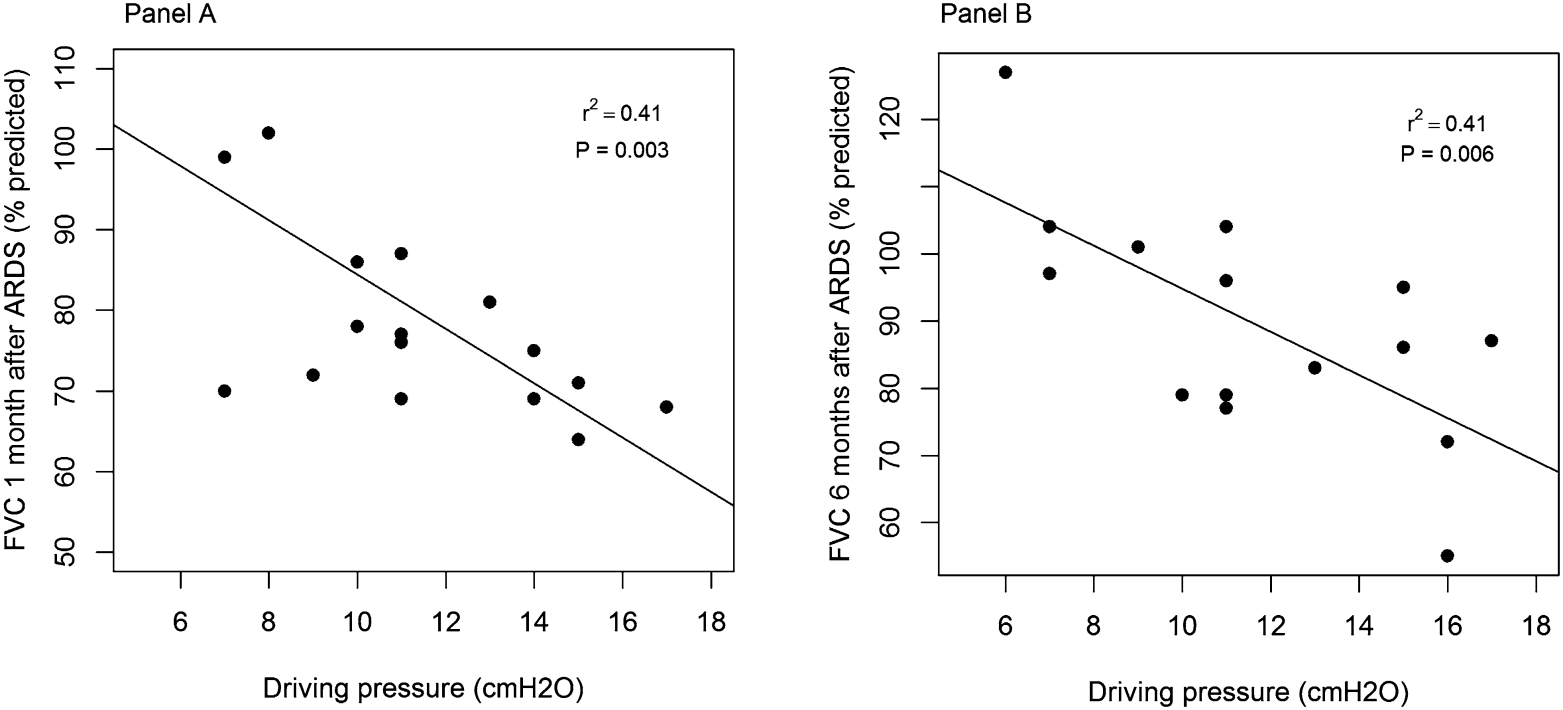
Relationship between driving pressure and forced vital capacity after 1 month (panel A) and 6 months (panel B) of acute respiratory distress syndrome

Driving pressure was the only ventilation variable significantly correlated with FVC at 1- (*r* = 0.65) and 6-month (*r* = 0.67) follow-up (Fig. 2). Driving pressure (*r* = 0.51) and APACHE II (*r* = 0.59) were correlated with DL_CO_ at month-1. Of note, tidal volume and respiratory system compliance were very weakly correlated with pulmonary function tests.

After testing for the confounding effects of tidal volumes, plateau pressures and baseline static respiratory compliance (*R*^2^ = 0.51, *F*(4,10) = 4.66, *p* = 0.022), the association between driving pressures and FVC at month-6 remained the only statistically significant one (*β* = − 4.62, IC95% (− 7.11 to − 2.13), *P* = 0.002).

When comparing the effects of the randomized treatments (OLA vs. ARDSnet) on FVC at month-6, there was a marginal difference between arms, with FVC of 4.54 ± 0.93 L versus 3.41 ± 1.04 L (OLA vs. ARDSnet, respectively, *P* = 0.06), representing 96 ± 20% versus 86 ± 15% of predicted, respectively (*P* = 0.32).

### CT scan analysis

Twenty-one patients were performed a HRCT at month-1 of follow-up. The median and interquartile range of the total lung volume (TLV) was 3.54 L (2.29–4.37); the total lung weight (TLW) was 1194 g (861–1873), and the mean pulmonary density (MPD) was 451 g/L (243–610). The P_15_ (percentile 15% for lowest lung densities) was 72 g/L (42–117).

At month-6, 16 patients were performed a HRCT. The median and interquartile range of TLV increased (*P* = 0.01) to 4.99 L (3.73–6.20), the TLW decreased (*P* = 0.01) to 762 g (652–934 g), the MPD decreased (*P* < 0.001) to 164 g/L (133–183), and P_15_ decreased (*P* = 0.02) to 49 g/L (23–65). Those longitudinal reductions in MPD, TLM and percent of nonaerated/poorly aerated lung volumes were all significant, as well as the increase in TLV.

Lung image parameters were significantly correlated with driving pressure and pulmonary function. After 6 months, individual MPD was significantly correlated with individual driving pressure (*r* = 0.53, *P* = 0.03), but not with respiratory system compliance (*P* = 0.95). Consistently, FVC at month-6 strongly correlated with CT parameters: MPD (*r* = − 0.86, *P* = 0.00005), % of nonaerated/poorly aerated volume (*r* = − 0.70, *P* = 0.003), TLV (*r* = 0.65, *P* = 0.008) and air/tissue volume ratio (*r* = 0.70, *P* = 0.003).

**Fig. 3:**
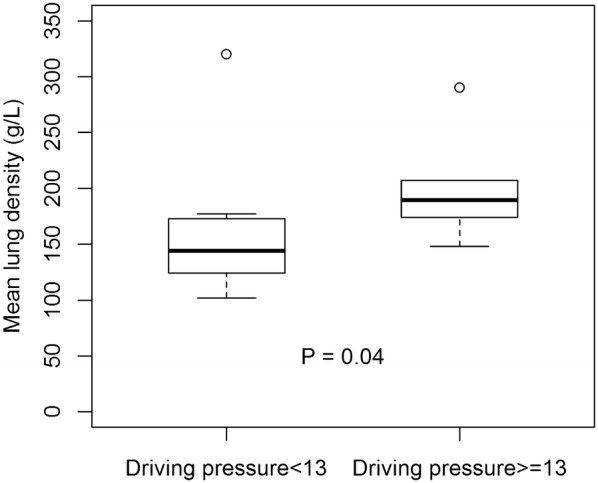
Mean pulmonary parenchyma density in the whole-lung CT scan in 17 ARDS patients after 6 months of follow-up split based on driving pressure median

Dividing patients based on median into high (HDP) or low (LDP) driving pressure groups (≥ 13 and < 13 cmH_2_O, respectively), MDP was higher in HDP group (*P* = 0.04; Fig. 3).

When comparing the effects of the randomized treatments (OLA vs. ARDSnet) on MPD at month-6, there was no difference, with MPD of 175 (131–176) g/L versus 171 (138–195) g/L (OLA vs. ARDSnet, respectively, *P* = 0.92). There was no difference in terms of TLV (*P* = 0.38) and TLW (*P* = 0.35).

### NT-PCP-III

From the 28 patients, 106 blood specimens were available for analysis of NT-PCP-III levels. All plasma samples had elevated levels, considering normal values, and considering the samples collected after 24 h from inclusion, after 3 days, and after 7 days or weaning time, the level increased over time (*P* = 0.03).

**Fig. 4:**
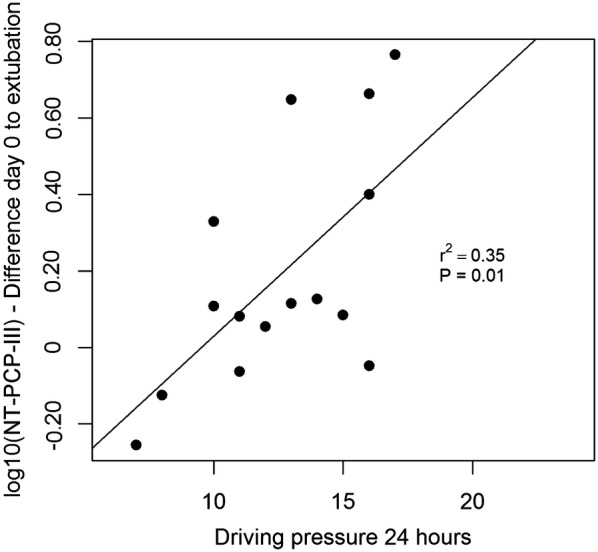
Relationship between driving pressure and log10 N-terminal-peptide type III procollagen (NT-PCP-III) difference between extubation day and day 0 of acute respiratory distress syndrome

Individual increments in log_10_NT-PCP-III levels, from baseline till extubation, correlated with individual values of driving pressures at 24 h (*β* = 0.006, IC95% (0.001–0.011), *r*^2^ = 0.35, *F*(1,13) = 8.55, *p* = 0.011; Fig. 4): the higher the driving pressure, the higher the increment in log_10_NT-PCP-III difference.

Mean pulmonary density (MPD) at month-6 was related to log_10_NT-PCP-III level during the first week of mechanical ventilation: at day 0 (*β* = 0.005, IC95% (0.001–0.010), *r*^2^ = 0.35, *F*(1,12) = 8.06, *p* = 0.014), day 1 (*β* = 0.006, IC95% (0.002–0.010), *r*^2^ = 0.34, *F*(1,14) = 8.84, *p* = 0.010), day 3 (*β* = 0.005, IC95% (0.0003–0.009), *r*^2^ = 0.25, *F*(1,12) = 5.38, *p* = 0.038).

**Fig. 5:**
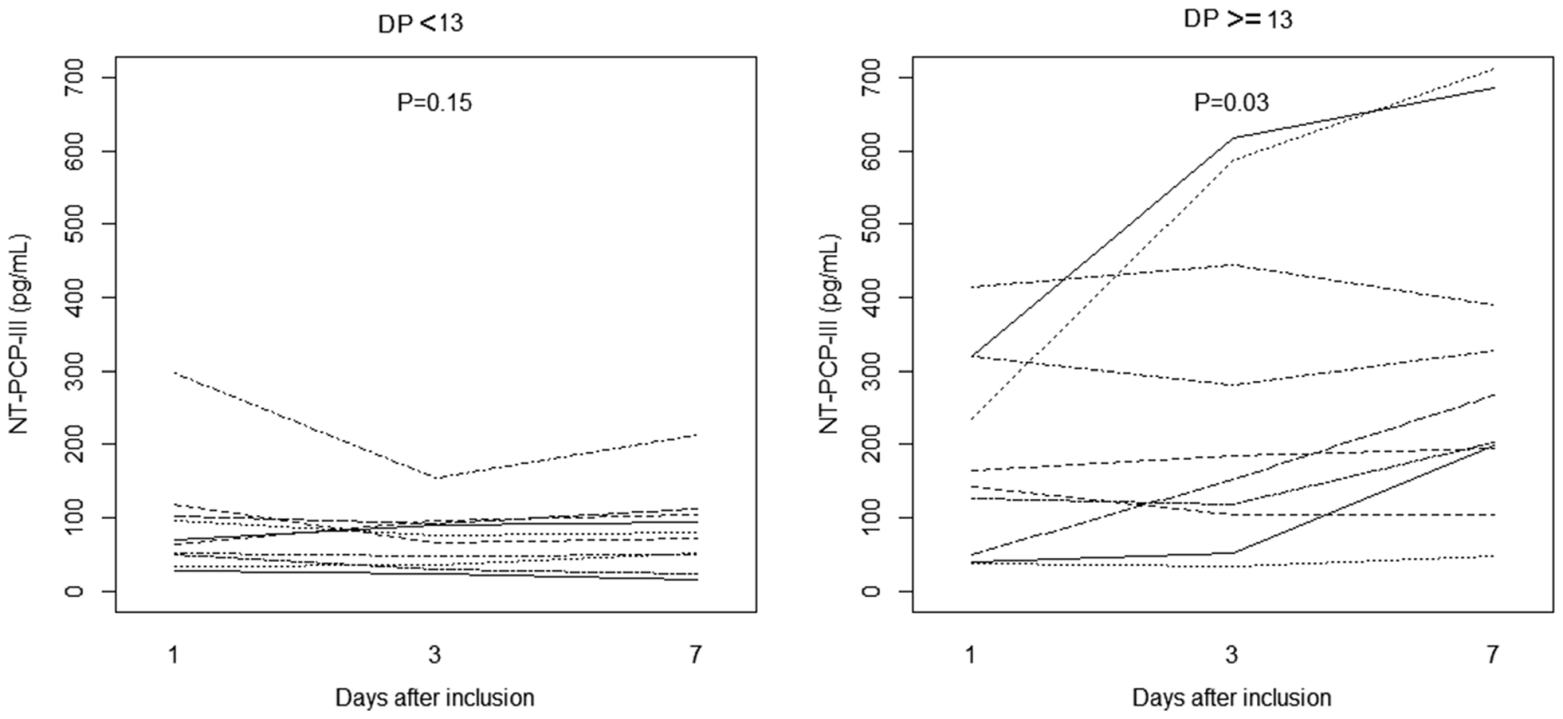
Changes in serum level of N-terminal peptide for type III procollagen over time in patients ventilated with driving pressure < 13 cmH_2_O 24 h after inclusion (*N* = 9) and patients ventilated with driving pressure ≥ 13 cmH_2_O 24 h after inclusion (*N* = 9). (NT-PCP-III = type III procollagen, 1 = day 1 after inclusion, 3 = day 3 after inclusion, 7 = level after extubation or at day 7 after inclusion)

Dividing patients into high (HDP) or low (LDP) driving pressure groups (≥ 13 and < 13 cmH_2_O, respectively), LDP group did not change levels over time (*P* = 0.15), while HDP group increased (*P* = 0.03; Fig. 5).

When comparing the effects of the randomized treatments (OLA vs. ARDSnet) on log_10_NT-PCP-III, there was no statistic significant difference at day 0 (*P* = 0.89), day 1 (*P* = 0.55), day 3 (*P* = 0.19) and on extubation (*P* = 0.65). There was a difference in terms of log_10_NT-PCP-III at day 7 (median log_10_NT-PCP-III 1.69 in OLA arm vs. 1.99 in ARDSnet, *P* = 0.03). There was no difference in the change between day 0 to day 1 (*p* = 0.69), to day 3 (*p* = 0.23), to day 7 (*p* = 0.31) and to extubation (*p* = 0.18).

### Other tests’ analysis

A standardized 6MWT was performed in 11 patients, but we could not observe any relationship between the walked distance and the ventilation variables selected. In terms of HRQoL, 10 patients completed SF-36 survey and we found a correlation between driving pressure (24 h after randomization) and the general health domain (*r* = − 0.69, *p* = 0.02).

## Discussion

In a population of patients surviving an episode of moderate to severe ARDS, there was a negative correlation between airway driving pressures, measured during the first 24 h of mechanical ventilation, and forced vital capacity (FVC) measured after 6 months of ARDS onset. This early measurement of driving pressure was representative of the applied strategy of protective ventilation, adjusted right after randomization and persisting at similar levels during the following days [20], and did not correlate with baseline compliance of the respiratory system. Also, we did not observe any relationship between tidal volumes (expressed in mL/PBW) and long-term pulmonary function tests.

Several studies involving ARDS survivors have shown a significant reduction in long-term lung function in a substantial proportion of patients, with a quarter of the patients presenting a FVC lower than 70% of predicted [5, 21]. In this subgroup of patients, the reduction in FVC was correlated with higher CT scores, but not with higher weakness score (acquired ICU weakness score), suggesting that the reduction in pulmonary function was related to lung fibrosis [6]. In fact, we observed an association between driving pressure and NT-PCP-III, a marker of fibrogenesis, paralleled by increased mean densities of the parenchyma, especially in those patients ventilated with higher driving pressure (Fig. 3). In those long-term studies, the changes in HRCT and in pulmonary function tests were both related to a poorer QoL [22]. In our study, the QoL could be further related to the level of driving pressures applied during mechanical ventilation.

Some investigators had previously correlated the total duration of mechanical ventilation and the levels of plateau pressure with the long-term results of pulmonary function tests and high-resolution CT studies, suggesting a relationship between mechanical ventilation and pulmonary dysfunction [6, 23]. This is the first time, however, that driving pressure was evaluated as a risk factor for long-term outcomes in survivors, and especially so after the general adoption of protective ventilation. In recent studies, driving pressures have been correlated with ARDS mortality, independently of tidal volume, PEEP and severity of illness, suggesting a causal role in the process of ventilator lung injury [24]. Commonly—as in this study—driving pressures and tidal volumes were weakly correlated, because lung compliance and ventilation strategies vary widely among patients [20]. Thus, we showed, similarly to the study of Amato et al. [20], that whereas a lower driving pressure was strongly correlated to better long-term outcomes, lower tidal volumes and higher baseline compliance (this latter reflecting lower severity of illness) were not.

A pro-fibrotic response to ARDS might be responsible for the physiologic abnormalities observed. Biomechanical interactions between cells and the extracellular matrix (ECM) proteins may be associated with the reorganization and remodeling of the ECM [25]. Collagen is the most important stress-bearing constituent of the parenchymal tissue and plays a critical role in mechanotransduction in lung repair and fibrosis development [26]. In the isolated rat lung or lung parenchymal strips, mechanical stretch resulted in enhanced NT-PCP-III gene expression, a by-product of type III collagen synthesis and a potential marker of collagen secretion [27, 28]. Interestingly, these studies suggested that the driving force (stress) applied to the tissue, but not the amplitude of resulting stretch, was responsible for collagen production.

Diffuse alveolar damage (DAD) is considered absent in approximately half of patients undergoing lung biopsy for nonresolving ARDS. The main alternatives to DAD were interstitial pneumonia and lung fibrosis, infections, cryptogenic organizing pneumonia and alveolar hemorrhage [29, 30]. In our study, the increase in NT-PCP-III, associated with higher mean lung densities and lower lung volumes, was very suggestive of an intense fibrosis. Similarly to patients with idiopathic pulmonary fibrosis, the intense remodeling concomitantly reduced the total lung weight and volume, with a progressive increase in lung densities—but not necessarily an increase in lung tissue mass [31]. The reductions in FVC in our patients were not accompanied by an increased residual volume, a finding that might otherwise suggest some weakness associated with severity of illness [32]. Thus, we believe that most of the reduction in FVC observed in our patients was related to fibrosis and remodeling of lung tissue.

In the general population, a low FVC has been associated with increased respiratory symptoms, functional limitation and mortality [33–35]. Thus, the observed relationship between driving pressure and long-term QOL might be mediated by the low FVC, secondary to lung fibrosis. Of note, some of our patients presented FVC values of less than 70% of predicted values, and studies have shown that even smaller changes in FVC can strongly influence the QoL.

## Limitations

The modest number of patients enrolled in a single center (representing 10% of the total number of patients included in the multicenter trial) may have compromised the power of this study to identify statistically significant relationships. The identified relationships, however, were consistent across different long-term variables, measured independently by CT, function tests and questionnaire, suggesting the strength of the association. The independent blood samples also added consistency to our results.

Another limitation of our study was the unknown functional status of the enrolled patients, who were lost during follow-up. Additionally, though patients with an exacerbation of previous lung diseases were excluded before randomization, preexisting lung disease may have interfered in the results, as patients were not evaluated by pulmonary function tests prior to enrollment.

Finally, driving pressure was measured at 24 h of the beginning of the protocol. Ideally, we should consider some average exposition during the whole mechanical ventilation period. In this study, however, similarly to the previous study of Amato et al. [20], the values of driving pressure measured at day-one were strongly correlated to the levels measured during the next days. We may not exclude, however, that additional adjustments in mechanical ventilation after 24 h of the protocol could also be associated with late pulmonary function and structure.

## Conclusion

In patients surviving after a moderate to severe ARDS there is an association between driving pressure measured 24 h after enrollment and lung function measured at 1 and 6 months after the ARDS onset, and this relationship was independent of tidal volume and independent of baseline compliance. These results suggest that, even in the context of protective tidal volume and plateau pressure, mechanical ventilation can still promote lung injury and fibrosis, highlighting the possible role of driving pressure in long-term outcomes.

## Abbreviations

ARDS: acute respiratory distress syndrome
HRQoL: health-related quality of life
NT-PCP-III: N-terminal-peptide type III procollagen
6MWT: 6-min walk test
QoL: quality of life
ICU: intensive care unit
COPD: chronic obstructive pulmonary disease
APACHE II: Acute Physiology and Chronic Health disease Classification System II
PEEP: positive end-expiratory pressure
PaO_2_: arterial partial pressure of oxygen
F_I_O_2_: inspired O_2_ fraction
ELISA: enzyme-linked immunosorbent assay
PFT: pulmonary function test
HRCT: high-resolution computed tomography
MPD: mean pulmonary density
TLV: total lung volume
TAV: total air volume
TLM: total lung mass
HU: Hounsfield unit
P15: threshold of 15%
SF-36: 36-item Short-Form General Health Survey
SD: standard deviation
*P*/*F*: PaO_2_/FIO_2_
*C*_stat_: static compliance
PBW: predicted body weight
OLA: open lung approach
DAD: diffuse alveolar damage
HDP: high driving pressure
LDP: low driving pressure
